# Cancer Prevention and Worksite Health Promotion: Time to Join Forces

**DOI:** 10.5888/pcd11.140127

**Published:** 2014-07-24

**Authors:** Cancer Prevention in the Workplace Writing Group

## Introduction

The workplace is recognized as a setting that can profoundly influence workers’ health and well-being (1,2). The Centers for Disease Control and Prevention (CDC) workplace health promotion efforts address cancer prevention by focusing on cancer screening programs, community–clinical linkages, and cancer risk factors (eg, tobacco use, physical inactivity) that also influence risk for other chronic diseases (http://www.cdc.gov/workplacehealthpromotion/). Some efforts focus specifically on cancer; some focus on general chronic disease prevention. Additionally, the National Institute for Occupational Safety and Health (NIOSH), part of CDC, provides research and recommendations to address workplace hazards posed by chemicals that may increase cancer risk (http://www.cdc.gov/niosh/topics/cancer/policy.html).

Existing resources can be leveraged to expand the scope of workplace initiatives to address additional cancer risk factors and disparities. Changes to the physical and social characteristics of work environments are likely to have greater impact than health education alone (3). Given the aging US population (which is expected to result in a marked increase in the number of cancer diagnoses over the coming decades) and the prevalence of numerous risk factors among working-aged adults (4,5), a multifaceted approach to cancer prevention in the workplace is timely and needed. In addition, community-based prevention efforts may offer unrealized opportunities to reach vulnerable working populations who are not served by workplace health promotion programs. In this essay, we draw attention to a wide variety of available CDC resources and provide ideas for new efforts to advance primary cancer prevention among working adults.

## Factors Related to Cancer Risk

Many cancer risk factors could be influenced through efforts targeting the work environment, including facilities, services, and policies. Table 1 shows examples of risk and protective factors that may be amenable to workplace interventions. Some examples are specific to the work environment, such as exposure to known carcinogens (eg, diesel exhaust), higher levels of which are often permitted in the workplace compared with the general environment. Others are behaviors (eg, tobacco use) or chronic conditions (eg, obesity) that may increase cancer risk. The table also provides examples of protective factors (eg, staying physically active) that may reduce cancer risk. These factors affect a high percentage of US workers, so even small changes could have a large impact at the population level. Many of the examples are also related to other health outcomes, and the benefits of addressing them would extend well beyond cancer prevention.

**Table 1:** Examples of Cancer Risk Factors and Protective Factors That May Be Amenable to Workplace Interventions^a^

Factors	Associated Cancers	Prevalence Among US Adults
**Risk factors**		
Alcohol consumption	Breast, colorectum, esophagus, larynx, liver, oral cavity, pharynx (6)	Approximately 55% of adults drink alcohol, 17% of adults are binge drinkers,^b^ and 6% are heavy drinkers (7).^c^
Asbestos	Larynx, lung, mesothelioma, ovary (8)	An estimated 1.3 million construction and general industry workers are potentially exposed to asbestos (9).
Diabetes	Bladder, breast, colon, endometrium, liver, pancreas (10)	Approximately 11% of adults have diabetes, and prevalence increases with increasing age (11).
Diesel exhaust	Lung (12)	The amount of diesel exhaust adults are exposed to varies greatly, but some of the highest levels of exposure are among certain types of workers (eg, truck drivers, miners).^d^
Obesity	Colorectum, endometrium, esophagus, gallbladder, kidney, pancreas, postmenopausal breast (6)	More than one-third of adults are obese (13).
Radiation exposure from medical imaging	Breast, leukemia, lung, thyroid (14)	Americans receive nearly half their total radiation exposure from medical imaging and other medical sources (15).
Radon	Lung (16)	Approximately 37% of all radiation exposure is attributed to radon, and there are an estimated 21,000 lung cancer deaths attributed to radon exposure each year (15,17).
Red meat consumption	Colorectum (6)	Adults consume an average of approximately 40 g of red meat per day per person (18).
Sedentary behavior	Colorectum, endometrium, ovary, prostate (19)	Among employed adults, 4 out of 5 have occupations that are sedentary or require only light physical activity (20).
Shift work	Breast (21)	Approximately 15% of full-time wage and salary workers usually work a nonstandard shift (22).
Tobacco use	Acute myeloid leukemia, bladder, cervical, colorectum, esophagus, kidney, larynx, liver, lung, oral cavity, pancreas, pharynx, stomach (23)	One in 5 working adults smokes cigarettes (24).
Ultraviolet radiation exposure	Skin (melanoma and nonmelanoma), melanoma of the eye (25)	Approximately 37% of adults experienced sunburn in the past 12 months (26); among employed adults, about 1 in 4 regularly works outdoors twice per week or more (27).
**Protective Factors**		
Fruit consumption	Esophagus, larynx, lung, pharynx, oral cavity, stomach (6)	Most adults do not meet federal dietary recommendations for fruit intake (28).
High-fiber diet	Colorectum (6)	Less than 5% of adults meet or exceed adequate intake levels for dietary fiber (29).
Physical activity	Colorectum, endometrium, postmenopausal breast (6)	Only 1 in 5 adults meets the 2008 Physical Activity Guidelines for Americans (30).
Radon testing and mitigation	Lung (16)	Only 3 states (Florida, New Hampshire, and Rhode Island) require radon testing in all public buildings (31).
Vegetable consumption	Esophagus, larynx, stomach, oral cavity, pharynx (6)	Most adults do not meet federal dietary recommendations for vegetable intake (28).
Weight maintenance	Postmenopausal breast (6)	Most adults experience clinically meaningful weight gain (≥22 pounds) during adulthood (32).

a This list is not intended to be exhaustive but rather to provide examples of cancer risk-related factors that could be addressed in workplace health promotion and protection efforts.

b Binge drinkers are defined as men having 5 or more drinks on 1 occasion and women having 4 or more drinks on 1 occasion.

c Heavy drinkers are defined as men having more than 2 drinks per day or more than 14 drinks per week and women having more than 1 drink per day or more than 7 drinks per week.

d The most recent estimates of occupational exposure are based on data from 1981–1983 and indicate that, at that time, approximately 1.35 million US workers were occupationally exposed to the combustion products of diesel fuel (http://www.cdc.gov/niosh/docs/88-116/#niosh1983).

## Integrating Health Protection and Health Promotion

Traditionally, workplace health promotion programs have focused on health-related behaviors (eg, tobacco use cessation), while health protection programs have focused on addressing safety and health risks and hazard mitigation. New research provides evidence that integrating these 2 approaches may enhance program effectiveness to improve employee health, safety, and well-being (2). As discussed in the National Prevention Strategy (http://www.surgeongeneral.gov/initiatives/prevention/strategy/), efforts to ensure worker safety on the job while also providing a work environment that supports healthy behaviors can create a culture in which worker health is viewed as a priority and healthy behaviors are more likely to be adopted by employees. NIOSH conducts research and develops programs to advance this integrated approach through the Total Worker Health program (http://www.cdc.gov/niosh/twh).

Because many cancers share risk factors with other diseases and chronic conditions, cancer prevention efforts in the workplace may also be enhanced by coordination with initiatives to address other diseases and chronic conditions. For example, the role of obesity as a risk factor for cancer and type 2 diabetes has been explored in occupational settings (33). This integrated and comprehensive approach maximizes program success and potential for sustainability over time.

## Linking With Community Resources

Given the “spillover” that often occurs between work and family lives (2,34), a systems approach addressing the interrelationship between work, family, and community can maximize benefits. For example, employers can partner with organizations in the surrounding community to offer health-related programs and services to employees that complement workplace interventions (http://www.cdc.gov/workplacehealthpromotion/assessment/assessment_interviews/index.html#5). These linkages can extend support to employees when outside the workplace and provide services that are beyond the capacity or expertise of the employer (eg, partnering with local fitness facilities to provide discounted memberships to employees, promoting local farmers markets for access to fresh produce) (35).

Public–private partnerships may also contribute to effectiveness by bringing specialized skills and resources to an intervention (36). Community linkages are particularly important for small and mid-sized employers for whom a lack of resources can be a barrier to implementation and sustainability.

## Disparities Across the Workforce

Differences across industries, workplace settings, and types of work create challenges to reaching certain groups and can contribute to cancer health disparities. For example, certain jobs expose workers to chemical and physical hazards (eg, second-hand smoke, excessive sun exposure) or require nonstandard work hours (eg, night shift work) that can contribute to an increased cancer risk (34). Furthermore, not all occupations are found in centralized work locations. Those working outside the typical office setting or without permanent worksites (eg, transportation and construction workers) may benefit from programs that are tailored to their work circumstances and link to other community resources. Additionally, telecommuting has become common among workers, and although the flexibility to telecommute may lend certain benefits, it may also create barriers to reaching employees through traditional worksite wellness programs (34,37).

Health disparities among low-wage and low-income (LW/LI) workers are particularly concerning, as these workers may lack certain protections and worksite benefits available to higher-earning workers and be ineligible for government antipoverty supports (38). Temporary contract workers, seasonal workers, and part-time workers earning low wages tend to face worse working conditions and receive fewer benefits than workers in more permanent positions (34). Women and racial/ethnic minorities are overrepresented among LW/LI workers, further exacerbating health disparities among these groups (34). Health promotion and protection efforts targeting LW/LI workers may help reduce disparities. Such efforts will require buy-in from employers and managers, and potential benefits to employers (eg, reductions in absenteeism) should be highlighted.

Almost half of cancer survivors, individuals who have been given a cancer diagnosis at some point in their lives, are of traditional working age (ie, 18–64 years) (http://cancercontrol.cancer.gov/ocs/prevalence/prevalence.html#age). Although the unique needs of cancer survivors are outside of the scope of this essay, workplace prevention efforts may also reduce the risk of second primary cancers or cancer recurrence among cancer survivors.

## CDC-Supported Program Activities

CDC’s National Comprehensive Cancer Control (CCC) Program supports states, tribes, and territories to establish coalitions, assess the burden of cancer, determine priorities, and develop and implement cancer plans (http://www.cdc.gov/cancer/ncccp/). A scan of activities conducted by CCC programs indicated that only 15 (22%) of 69 programs are engaged in efforts to promote primary cancer prevention in the workplace (J. Townsend, A. Neri, MD, oral communication, July 2013). CCC activities targeting the workplace include general adoption of worksite policies and provision of information about physical activity and nutrition. Some programs are seeking to expand existing prevention-related policies to reach more community members through the workplace setting; others are focused on specific worksites (eg, sun-safety at ski resorts, tobacco exposure in casinos). Identifying low-cost strategies and making them easily available may facilitate further adoption. CCC grantees also provide a strong network for information dissemination through their coalitions as new resources become available.

NIOSH conducts research and develops programs to improve workplace conditions and practices to lower the incidence of occupationally related cancers. NIOSH focuses primarily on industrial sectors, occupations, and populations with elevated risk through basic bench research, on-site health hazard evaluations, recommendations for occupational exposure limits, timely health alerts, and training programs.

## CDC-Supported Resources

Many CDC-supported workplace health promotion and protection resources are available (Table 2). For example, CDC provides guidance on smoke- and tobacco-free workplace policies that reduce tobacco use, initiation of tobacco use, and secondhand smoke exposure. Although not necessarily framed in the context of cancer prevention, many other CDC-supported resources address physical inactivity, diet and nutrition, and tobacco use, and some address cancer screening and early detection and weight management. Concerted efforts to share these resources with CCC programs and other funded partners may increase their dissemination and use at the state and local levels. Additionally, the CDC-supported Guide to Community Preventive Services provides evidence-based recommendations for worksite health promotion strategies (http://www.thecommunityguide.org/worksite/index.html). Continuing to link CCC grantees with relevant resources and expertise available in other parts of CDC may help maximize the impact of CDC’s prevention work targeting adults.

**Table 2:** Centers for Disease Control and Prevention (CDC) Resources Available to Promote Cancer Prevention in the Workplace

Resource/Goal or Purpose	Target Audience	Content	Cancer-Related Factors Addressed
**Community Health Resources: Worksite Wellness,** Community Health and Program Services Branch, http://apps.nccd.cdc.gov/dach_chaps/Default/LinksHealthTopic.aspx?topic=26			
Assistance for community health partners, coalitions, and activists as they navigate CDC’s Web-based resources	Employers, employees, and worksite health promotion planners	Searchable database; communications and marketing campaigns; cross-cutting programs; data and statistics; guidelines and recommendations; policy, partnership, and planning tools	Tobacco use; diet and nutrition; physical activity; weight management; screening and early detection
**Essential Elements of Effective Workplace Programs and Policies for Improving Worker Health and Wellbeing,** National Institute for Occupational Safety and Health, http://www.cdc.gov/niosh/TWH/essentials.html			
Guide for employers and employer–employee partnerships wishing to establish effective comprehensive work-based health protection and health promotion programs that sustain and improve worker health	Employers, employees, and worksite health promotion planners	Framework of 20 components categorized by organizational culture and leadership, program design, program implementation and resources, and program evaluation; guiding principles; practical direction	Tobacco use; diet and nutrition; physical activity; weight management
**Healthier Worksite Initiative,** Division of Nutrition, Physical Activity, and Obesity, http://www.cdc.gov/nccdphp/dnpao/hwi/index.htm			
Comprehensive one-stop–shop resource for workforce health promotion program planners	Worksite health promotion planners in state and federal government	Resources on a wide variety of workforce health promotion topics; program design tools and information about program planning and needs assessments; basic information about policies that affect health promotion at federal workplaces that also explains why they are important; resources and links to documents and websites with credible, useful information	Tobacco use; diet and nutrition; physical activity; weight management; screening and early detection
**Healthy Hospital Choices,** Division of Nutrition, Physical Activity, and Obesity, http://www.cdc.gov/nccdphp/dnpao/hwi/docs/HealthyHospBkWeb.pdf			
Summary of meeting and recommendations for action from expert panel on policy and environmental approaches to improve hospital worksite wellness	Hospital employers, hospital administrators, and public health practitioners	15 recommendations to improve the physical activity, nutrition, and tobacco-free environments of hospitals for their employees, patients, and visitors	Tobacco use; diet and nutrition; physical activity
**Investing in Health Guide,** Partnership for Prevention in collaboration with CDC, http://www.prevent.org/Worksite-Health/Investing-in-Health-Workplace-Guide.aspx			
Guide to provide employers with guidance that can improve employee health	Employers	Easy-to-follow action steps translated from evidence-based recommendations from the US Preventive Services Task Force and the Task Force on Community Preventive Services; guide to help employers manage health care spending and improve employee morale	Tobacco use; diet and nutrition; physical activity; screening and early detection
**Leading by Example,** Partnership for Prevention in collaboration with CDC, http://www.prevent.org/initiatives/leading-by-example.aspx			
Peer-to-peer communication campaign to educate chief executive officers about the benefits of worksite health promotion and encourage employers to adopt effective practices to improve employee health	Chief executive officers, upper-level management, and employers	Publications to leverage the workplace to improve health by promoting greater business involvement in health promotion and disease prevention; publications to help chief executive officers influence the American health care system to emphasize prevention rather than treatment	Tobacco use; diet and nutrition; physical activity; screening and early detection
**Lean *Works!*,** Division of Nutrition, Physical Activity, and Obesity, http://www.cdc.gov/leanworks/			
Free Web-based resource to help employers respond to the obesity epidemic	Employers and worksite health promotion planners	Interactive tools; evidence-based resources; obesity cost calculator	Diet and nutrition; physical activity; weight management
**National Healthy Worksite Program,** Division of Population Health, http://www.cdc.gov/nationalhealthyworksite/index.html			
Program designed to assist employers in implementing science-based and practice-based prevention and wellness strategies that will lead to specific, measureable health outcomes to reduce chronic disease rates	Employers and worksite health promotion planners	Stepwise approach to create or improve a workplace health program; comprehensive tool kit to support training as well as supporting materials and guidance for employers; community and national training opportunities; webinars and teleconferences; on-going evaluation	Tobacco use; diet and nutrition; physical activity; weight management
**Promising Practices for Total Worker Health (TWH),** National Institute for Occupational Safety and Health, http://www.cdc.gov/niosh/TWH/practices.html			
Quarterly feature in the electronic newsletter *TWH in Action!* to describe program design, frameworks, and implementation and to serve as a resource for understanding components of effective integrated safety and health programs	Employers, workers, human resource professionals, workplace safety and health professionals, and worksite health promotion professionals	Case examples that demonstrate characteristics of employer programs that aim to integrate health protection and health promotion	Tobacco use; diet and nutrition; physical activity; weight management; long work hours, fatigue, shift work, and healthy sleep
**Save Lives, Save Money: Make Your Business Smoke-Free,** Office on Smoking and Health, http://www.cdc.gov/tobacco/basic_information/secondhand_smoke/guides/business/			
Brochure that provides information on exposure to secondhand smoke in the workplace and the benefits to employers once a smoke-free workplace has been implemented	Employers	Informational brochure	Tobacco use
**School Employee Wellness: A Guide for Protecting the Assets of Our Nation’s Schools,** Directors of Health Promotion and Education in collaboration with CDC, http://www.healthyschoolsms.org/staff_health/documents/EntireGuide.pdf			
Comprehensive guide that provides information, practical tools, and resources for school employee wellness programs	School district policy makers, school administrators, and employees	Resources to promote the benefits of school employee wellness programs; model for establishing, implementing, and sustaining a school employee wellness program; practical tools and resources to support the implementation of school employee wellness programs	Tobacco use; diet and nutrition; physical activity; weight management; screening and early detection
**Steps to Wellness,** Division of Nutrition, Physical Activity, and Obesity, http://www.cdc.gov/nccdphp/dnpao/hwi/toolkits/pa-toolkit.htm			
Guide to implementing the *2008 Physical Activity Guidelines for Americans in the Workplace*, provides easy and understandable steps on how to increase the physical activity of employees in the workplace	Employers and worksite health promotion planners	A document explaining why businesses should create a culture that values physical activity for its employees; steps to take to promote physical activity and create a culture of wellness in the workplace; tools and templates to help businesses plan, promote, and implement workplace physical activities; additional resources about physical activities and worksite wellness programs	Physical activity
**Swift Worksite Assessment and Translation,** Division of Nutrition, Physical Activity, and Obesity, http://www.cdc.gov/nccdphp/dnpao/hwi/programdesign/swat.htm			
Evaluation method to rapidly assess and identify promising practices in workplace health promotion programs	Employers	Site identification and selection; 2-day site visit; post-visit assessment of promising practices; evaluation capacity-building; translation and dissemination	Diet and nutrition; physical activity; weight management
**Workplace Health Promotion Portal,** Division of Population Health, http://www.cdc.gov/workplacehealthpromotion/			
Online tool kit for active approaches to improving employee health	Employers and worksite health promotion planners	Wide variety of information, tools, resources, and guidance for employers; searchable database by topics of interest; assessment, planning, workplace governance, implementation, and evaluation modules	Tobacco use; diet and nutrition; physical activity; screening and early detection
**Worksite Health ScoreCard**, Division for Heart Disease and Stroke Prevention, http://www.cdc.gov/dhdsp/pubs/worksite_scorecard.htm			
Tool to help employers assess whether they have implemented evidence-based health promotion interventions or strategies in their worksites	Employers and worksite health promotion planners	12 topic sections; valid and reliable assessment tool for comprehensive health promotion and disease prevention programs; identification of program gaps and prioritization of high-impact strategies	Tobacco use; diet and nutrition; physical activity; weight management; screening and early detection

## Conclusion

The workplace is a key setting for efforts to reduce cancer risk among adults. Although some cancer risk factors have garnered attention in worksite health promotion, others have been somewhat overlooked and may be worth considering for future interventions. More could be done to take advantage of existing resources and prevention networks. Efforts to promote cancer prevention in the workplace may need to take an integrated and comprehensive approach by addressing individual behaviors, organizational culture, policies, and other environmental factors that influence cancer risk. We hope that the information and resources described in this essay will be useful to those working in health promotion and cancer prevention programs targeting adults.
